# Label-Free Plasmonic Biosensor for Rapid, Quantitative, and Highly Sensitive COVID-19 Serology: Implementation and Clinical Validation

**DOI:** 10.1021/acs.analchem.1c03850

**Published:** 2021-12-31

**Authors:** Olalla Calvo-Lozano, Miquel Sierra, Maria Soler, Maria Carmen Estévez, Luis Chiscano-Camón, Adolfo Ruiz-Sanmartin, Juan Carlos Ruiz-Rodriguez, Ricard Ferrer, Juan José González-López, Juliana Esperalba, Candela Fernández-Naval, Leticia Bueno, Ruben López-Aladid, Antoni Torres, Laia Fernández-Barat, Sarah Attoumani, Rémi Charrel, Bruno Coutard, Laura M. Lechuga

**Affiliations:** † Nanobiosensors and Bioanalytical Applications Group (NanoB2A), Catalan Institute of Nanoscience and Nanotechnology (ICN2), CSIC, CIBER-BBN and BIST, Campus UAB, Bellaterra, Barcelona 08193, Spain; ‡ Intensive Care Department, Vall d’Hebron Hospital Universitari, Vall d’Hebron Barcelona Hospital Campus, Passeig Vall d’Hebron 119-129, Barcelona 08035, Spain; § Shock, Organ Dysfunction and Resuscitation Research Group, Vall d’Hebron Research Institute (VHIR), Vall d’Hebron Hospital Universitari, Vall d’Hebron Barcelona Hospital Campus, Passeig Vall d’Hebron 119-129, Barcelona 08035, Spain; ∥ Clinical Microbiology Department, Vall d’Hebron Hospital Universitari, Vall d’Hebron Barcelona Hospital Campus, Passeig Vall d’Hebron 119-129, Barcelona 08035, Spain; ⊥ Vall d’Hebron Institut de Recerca (VHIR), Vall d’Hebron Barcelona Hospital Campus, Passeig, Vall d’Hebron 119-129, Barcelona 08035, Spain; # Department of Genetics and Microbiology, Universitat Autònoma de Barcelona, Plaça Cívica, Bellaterra, Barcelona 08193, Spain; ∇ Cellex Laboratory, CiberRes (Centro de Investigación Biomédica en Red de Enfermedades Respiratorias, 06/06/0028), Institut d’Investigacions Biomèdiques August Pi I Sunyer (IDIBAPS), Carrer de Roselló 149, Barcelona 08036, Spain; ○ School of Medicine, University of Barcelona, Carrer de Casanova, 143, Barcelona 08036, Spain; ◆ Department of Pneumology, Thorax Institute, Hospital Clinic of Barcelona, Carrer de Villarroel, 170, Barcelona 08036, Spain; ¶ Unité Des Virus Émergents (UVE: Aix-Univ-IRD 190-Inserm 1207), Marseille 13005, France

## Abstract

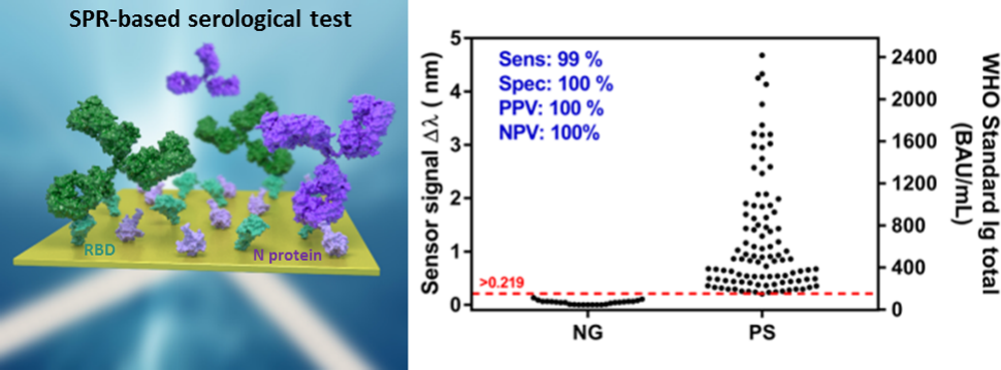

Serological tests are essential for the control and management of COVID-19 pandemic (diagnostics and surveillance, and epidemiological and immunity studies). We introduce a direct serological biosensor assay employing proprietary technology based on plasmonics, which offers rapid (<15 min) identification and quantification of severe acute respiratory syndrome coronavirus 2 (SARS-CoV-2) antibodies in clinical samples, without signal amplification. The portable plasmonic device employs a custom-designed multiantigen (RBD peptide and N protein) sensor biochip and reaches detection limits in the low ng mL^–1^ range employing polyclonal antibodies. It has also been implemented employing the WHO-approved anti-SARS-CoV-2 immunoglobulin standard. A clinical validation with COVID-19 positive and negative samples (*n* = 120) demonstrates its excellent diagnostic sensitivity (99%) and specificity (100%). This positions our biosensor as an accurate and easy-to-use diagnostics tool for rapid and reliable COVID-19 serology to be employed both at laboratory and decentralized settings for the disease management and for the evaluation of immunological status during vaccination or treatment.

It has been over a year since the World Health Organization (WHO) declared COVID-19 as a pandemic. The outbreak of this infectious disease, which likely originated in the Hubei region (China) in December 2019 caused by the SARS-CoV-2 virus (severe acute respiratory syndrome coronavirus 2), has rapidly spread worldwide, and it is generating unprecedented and devastating consequences at health, social, and economic levels. To date, COVID-19 has affected more than 276 million people, with more than 5 million deaths.^1^ The emergence of an unknown virus with a lack of population’s immunity and accurate diagnostic methods, together with the disease peculiarities (i.e., varied symptomatology or asymptomatology in a significant percentage of the infected people, long incubation times, high transmission rate, and so forth), have undoubtedly contributed to ease its unnoticeable spread and hinder a fast and early detection of many cases.^2−4^

Current standard diagnosis for the detection of an active infection relies on the detection of the SARS-CoV-2 viral genetic material from respiratory samples, mainly by RT-PCR (reverse transcription polymerase chain reaction),^5,6^ which provides excellent levels of sensitivity and specificity, but requiring centralized and specialized laboratories, and between 3 and 48 h to deliver results. To overcome its limitations related to long turnaround times, rapid antigen tests have already been developed and are being employed in many countries as the point-of-care test, although their sensitivity and reliability do not reach yet those achieved with genomic molecular assays.^7,8^ Complementary to the detection of the active infection, serological tests, which detect the presence of immunoglobulins (Ig) in blood generated by the infected host, play an important role in infectious disease surveillance and pandemic management, providing relevant information to estimate the prevalence of the virus and to better understand the dynamics of acquired immunity. In the case of SARS-CoV-2, the immune response is soon triggered, and antibodies are detectable after a few days postinfection. First, IgMs appear during the acute infection phase, which decline with time after a few days or even months. Then, long-lasting IgGs are generated, as well as IgA antibodies. IgGs are expected to remain in the blood stream at significant concentrations for at least months after infection, conferring immunity to the virus.^9,10^ Although serology assays are not suited for systematic detection of the virus, they are very helpful for the diagnosis of past infections (indirect testing) of suspected patients with negative PCR results, in the identification of asymptomatic patients, and also during the development of new vaccines or treatments.^9,11,12^ In addition, serological tests are extremely useful in hospitals for ICU bed management and the deisolation of post COVID-19 patients (i.e., PCR-positive patients with a positive serological test). Finally, the emergence of SARS-CoV-2 variants with increased resistance to sero-neutralization by antibodies induced after vaccination or primary infection makes serological tests a key component for the response to these variants. The serological assays developed for COVID-19 are based on the identification of IgMs and IgGs (and, to a less extent, IgAs), which are specific for most abundant viral antigens, including the spike protein [S1 and S2 subunits, and the receptor-binding domain (RBD)] and the nucleocapsid N protein. Traditional microplate-format immunoassays, such as ELISA (enzyme-linked immunosorbent assay) and CLIA (chemiluminescence immunoassay), are widely used in clinics, as they provide high sensitivities, can be automated, and offer multiplexed capabilities, but they require specific equipment and trained personnel in dedicated laboratories and can be time consuming because of sample manipulation and/or long incubation times.^13^ For massive screening, immunochromatographic lateral flow assays (LFA) have been widely spread because of its facile handling and rapid time-to-result response, becoming the most commercialized assays to perform SARS-CoV-2 serology tests. Some of them can differentiate the type of antibody (IgG and/or IgM) and thus provide information regarding the stage of the infection (e.g., acute phase or past infection), but only in a qualitative manner. Although they provide fast results (15 min assay) at the point-of-care (POC), some recent studies show that they are not reliable and accurate enough because of their moderate sensitivity (90–94%).^14−16^ The development of serological assays capable of performing quantitative analysis is critical for some potentially useful scenarios.^17^ These scenarios include monitoring acquired immunity over time, to be able to predict the duration of acquired immunity, evaluate seroconverted patients’ plasma for potential reinfusion in other patients, manage hospital beds and COVID-19 patient isolation, understanding the relationship between antibody levels and the severity of the symptoms, to carry out large-scale epidemiology studies for COVID-19 incidence determination, or helping in vaccine development.^11,18,19^

The ongoing pandemic situation, thus, demands advanced analytical tools that overcome aforementioned sensitivity limitations in serology testing, while still facilitating fast, quantitative, and reliable detection at the POC. Optical biosensors are well-positioned to fulfill these needs as they are sensitive techniques capable of performing label-free, direct, and quantitative analysis. Plasmon-based technologies, as the surface plasmon resonance (SPR) biosensor, offer remarkable performance and versatility, and they have become one of the most consolidated biosensor technologies for biomolecular interactions and clinical diagnostics,^20,21^ with potential for compactness and miniaturization. Moreover, SPR biosensing have been applied for multiple clinical applications in virology, including serological assays related to dengue virus,^22,23^ Salmonella,^24^ Epstein–Barr virus,^25^ and also for the first SARS-CoV.^26^ A few preliminary studies and perspectives have been recently reported as well for SARS-CoV-2,^27−29^ advocating for the potential of this technology as POC diagnostic devices.

We have fully implemented an SPR-based serological test combining RBD and N viral antigens for the detection of SARS-CoV-2-specific antibodies from human sera (Figure 1). Our SPR biosensor offers label-free and real-time monitoring of biomolecular interactions, therefore enabling a one-step quantitative serological assay performed in less than 15 min, including sample injection, signal readout, and result interpretation. After an in-depth optimization of the biorecognition interface and bioassay conditions, we have achieved analytical sensitivity levels in the range of ng mL^–1^, comfortably below the estimated antibody levels in patients, which appear to be in the μg mL^–1^ range.^30^ In order to validate our technology, we performed a comprehensive clinical validation with COVID-19-positive and -negative samples collected from patients attended in different hospitals, comparing our results to standard and regulated techniques (i.e., ELISA and CLIA), as well as commercial rapid tests based on LFA.

**Figure 1:**
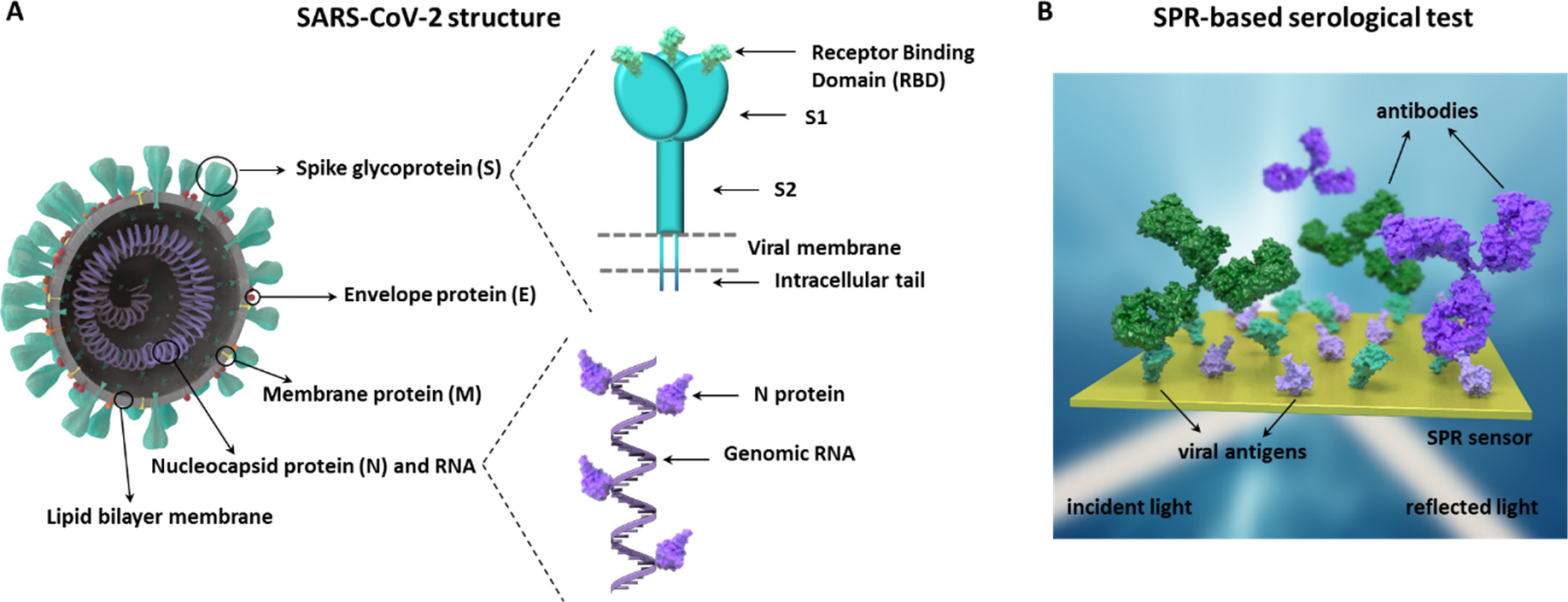
**(A)** SARS-CoV-2 virus structure and details of the spike (S) and nucleocapsid (N) proteins; **(B)** scheme of the two-antigen coimmobilized sensor biochips employed in the SPR biosensor for COVID-19 serology.

## Experimental Section

### SPR Biosensor Device

The biosensor device employed is a homemade designed and assembled SPR that incorporates all the optical and microfluidic components in a compact and user-friendly platform (20 × 20 cm^2^). A description of the device is provided in the Supporting Information (SI) and in Figure S1. All the experiments were carried out in appropriate safety facilities, with the SPR biosensor located in a laboratory of biosafety level 2 (BSL-2).

### Antibody Detection Assays

The experiments were performed with chips immobilized with the viral proteins (N protein, RBD peptide, or N + RBD 1:1, prepared as described in the Supporting Information) and two different polyclonal antibodies, pAb-N and pAb-RBD, specific for N protein and the RBD domain, respectively, and with the first WHO international standard anti-SARS-CoV-2 human immunoglobulin.

Real-time sensorgrams generated during the injection of the antibodies (100 μL) were obtained in all the cases, monitoring the specific binding in each case (i.e., shift in the position of the resonance peak (Δλ, nm) to higher wavelengths). For single-antigen gold sensor chips, calibration curves were generated by analyzing different concentrations of the corresponding specific antibody (ranging from 100 ng mL^–1^ to 10,000 ng mL^–1^) in standard buffer (PBST + DS) or in commercial serum diluted to 10%. For the RBD/N coimmobilized sensor chips, several mixtures of pAb-RBD and pAb-N antibodies (1:1) at equal concentrations (from 100 ng mL^–1^ to 10,000 ng mL^–1^) were prepared in serum and analyzed after diluting at 10%. Calibration curves were also generated employing the first WHO-approved standard for serology assays, consisting of freeze-dried pooled plasma from eleven patients recovered from COVID-19 disease, whose stock solution has an assigned arbitrary unit of 1000 BAU mL^–1^ (BAU, binding antibody units). Several concentrations were analyzed (ranging from 1.25 to 500 BAU mL^–1^) in standard buffer (PBST + DS) or in commercial serum diluted to 10% on the RBD/N coimmobilized sensor chip. All the antibody solutions were injected over the sensor chip at a constant flow of 15 μL min^–1^. In all the cases, antigen–antibody interaction was disrupted by injecting a 20 mM NaOH regeneration solution during 1 min at constant flow rate. Antigen-biofunctionalized plasmonic sensor chips could be reused between 15 and 20 times without altering or modifying the immobilized proteins and the assay performance.

### Clinical Sample Collection

A total of 125 clinical samples were collected from two hospitals in Barcelona (Spain) in three different batches. Two batches were provided by Vall d’Hebron University Hospital (VH.1 *n* = 15, and VH.2 *n* = 70), and a third batch was provided by the Clinic Hospital of Barcelona (CH.1 *n* = 40). Details on sample and data collection are summarized in the Supporting Information.

### Data Analysis

The real-time sensorgrams were processed extracting the final response (Δλ) after signal stabilization once the whole sample volume has passed through the flow cell. For the flow rate employed and the sample volume, this corresponds to approximately 1000 s after injection. Details on the fitting curves and to extract detection assay characteristics and statistical analysis are described in the Supporting Information.

## Results and Discussion

### Biosensor Assay Development and Analytical Characterization

The in-house-developed SPR biosensor platform employed monitors the shift in the position of the resonance peak of the plasmonic sensor chips, which reflects binding (Δλ > 0) or desorption events (Δλ < 0), the signal being proportional to the number of events (i.e., concentration). Our biosensor device has previously demonstrated its potential for clinical diagnostics in several areas,^31–36^ including infectious diseases,^32^ and also for the direct detection of antibodies in human serum,^31,34^ enabling a one-step, label-free, and sensitive and reliable detection. These features are crucial to develop a fast test with response times below 15–20 min (thanks to the no-need of secondary reagents or further signal amplification steps), and with the potential of providing quantitative information (i.e., the concentration range of antibodies in serum).

**Figure 2:**
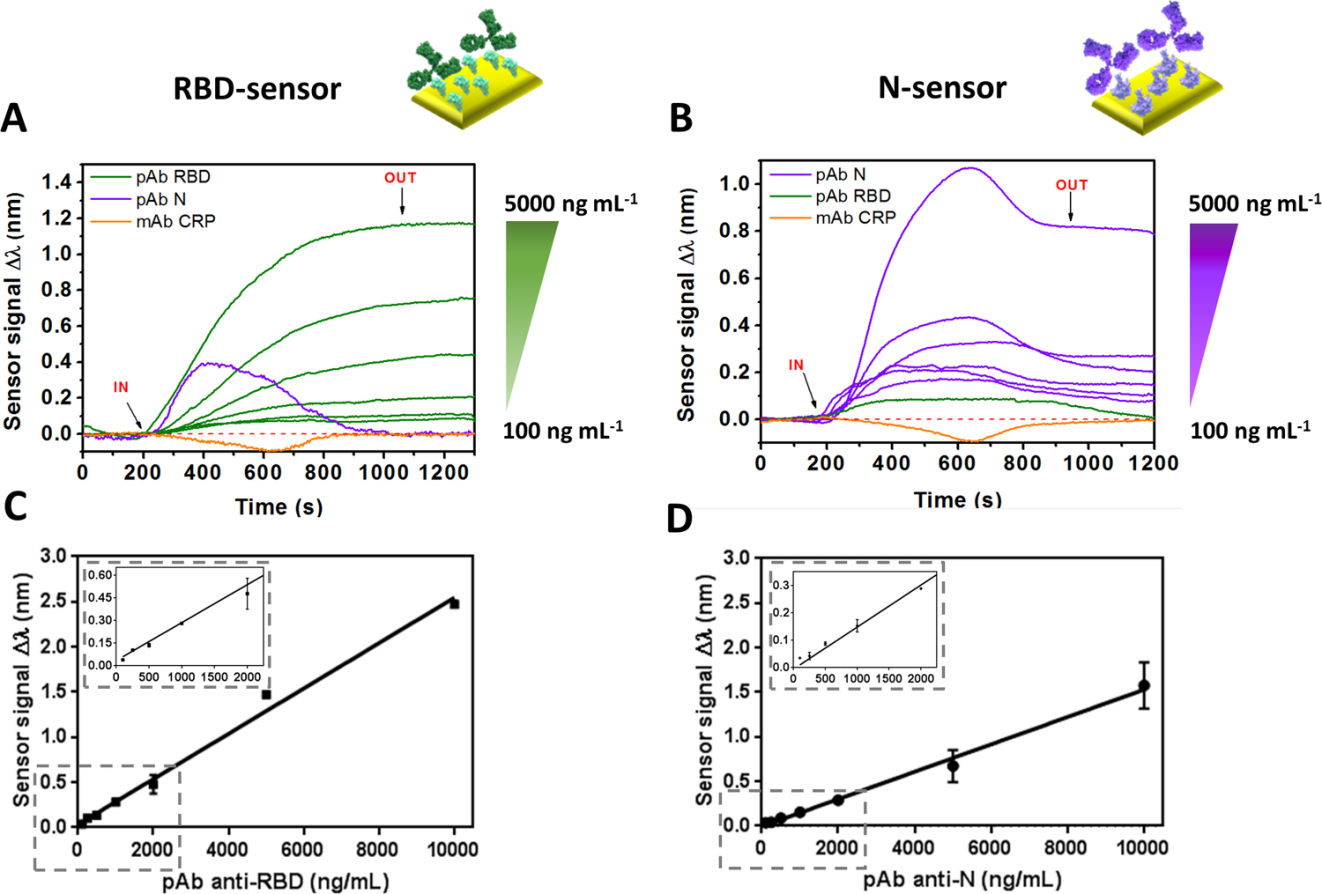
Real-time sensorgrams for different antibody concentrations over a **(A)** RBD-coated sensor chip and **(B)** N-coated sensor chip, in standard buffer conditions. Calibration curves in standard buffer for **(C)** RBD-coated sensors chips and **(D)** N-coated sensor chips, using the corresponding pAb. Each signal corresponds to the mean ± SD of triplicate measurements. Nonspecific antibodies were measured at a concentration of 2000 ng mL^**–1**^. IN (time ∼ 200 s) and OUT (time ∼ 1100 s) arrows indicate the start and end time of the injection, respectively.

In the case of COVID-19 serological assays, a key aspect to maximize both specificity and sensitivity is the viral antigen used for the detection of the antibodies. The N protein and the RBD peptide contained in the spike protein appear to be both specially highly specific targets.^9,13,37,38^ Thus, we developed two different biofunctionalized sensor chips employing the N and RBD antigens in order to capture the antibodies generated by the host. To evaluate the performance of the biosensor-based assay, we employed commercial polyclonal antibodies for both N and RBD antigens, which can mimic the pool of antibodies with different antigen affinities produced by a host individual after infection. Figure 2A,B show representative detection signals (i.e., real-time sensorgrams) obtained for the two different biofunctionalized surfaces (i.e., N and RBD) with different pAb concentrations in buffer, gradually increasing as the concentrations were higher (i.e., Δλ obtained after signal stabilization).

A direct and linear relationship between the antibody concentration and the signal was observed (see Figure 2C,D) for the range of antibodies analyzed (i.e., from 100 to 10,000 ng mL^–1^), being possible to determine the limit of detection (LOD, defined as the concentration corresponding to a blank signal plus three times its standard deviation) in both cases: 19.9 ng mL^–1^ for anti-RBD immunoassay (slope = 0.2511 nm mL μg^–1^, *R*^2^ = 0.992) and 45.6 ng mL^–1^ for anti-N (slope = 0.1536 nm mL μg^–1^, *R*^2^ = 0.994). Some studies suggest that the antibody concentrations in COVID-19 patients’ serum might lie in the range of μg mL^–1^.^30^

According to these values, the performance of our biosensor provides enough analytical sensitivity for COVID-19 serological testing with both RBD and N-coated sensor chips. The specificity was also evaluated in order to assure the absence of nonspecific interactions of antibodies with the sensor chip surface. As Figure 2A,B shows, neither pAb-N nor pAb-RBD interacted with the opposite antigen surface (i.e., net sensor response after signal stabilization Δλ = 0 nm), proving that no cross-reactivity between the antigen–antibody pairs was taking place. Similarly, a SARS-CoV-2 nonrelated antibody (i.e., anti-CRP) did not result in any signal, overall, confirming that the signals come exclusively from specific antigen–antibody interactions.

In order to apply the described methodology in serological assays and therefore in patient’s sera samples, we had to take into account the influence of the serum matrix on the sensor surface and the recognition event, as undiluted serum contains high amounts of proteins and other compounds that could generate nonspecific interactions or hinder the protein–antibody interaction. For this reason, we decided to employ a combination of blocking agents, including poly-l-lysine-grafted poly(ethylene glycol) (PLL-*g*-PEG), detergent Tween 20, and dextran sodium sulfate (DS), all of which have successfully reduced nonspecific interactions in previous studies.^34,35^

**Figure 3:**
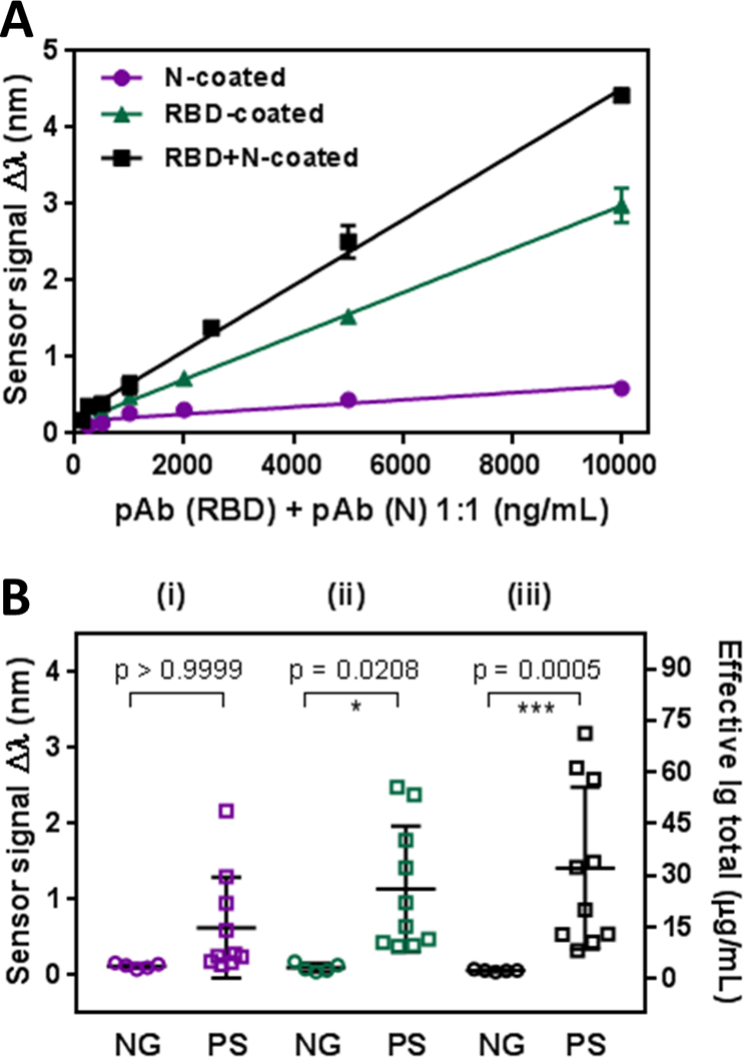
(A) Calibration curves with pAb-N and pAb-RBD in 10% diluted commercial serum using three different biofunctionalized surfaces (N, RBD and RBD + N). Sensor response represents the mean ± SD of three measurements. (B) Statistical comparison between the positive (PS) and negative (NG) clinical samples: (i) N-coated sensor chips; (ii) RBD-coated sensor chips; (iii) RBD + N-coated sensor chip. Kurskal–Wallis test (*p* = 0.05). Total Ig concentration calculated from the WHO standard anti-SARS-CoV-2 immunoglobulin calibration curve is shown in the right axis.

The performance of the assays in serum was directly evaluated with serum diluted at 10%, as the detectability range of the assay might certainly tolerate this dilution (i.e., LOD in the ng mL^–1^ range and presumably expected Ig concentrations in the μg mL^–1^ level) and still ensure a reliable semiquantitative and quantitative detection. This dilution factor is considerably lower than the one commonly employed in ELISA or CLIA tests, which is around 40–200 times.^30,39^ In fact, under these conditions, no undesired effects were observed for commercial serum, with negligible nonspecific adsorptions and with a similarly wide dynamic range (see Figure 3A). The limit of detection achieved for the N biofunctionalized surface was twice higher than that under standard buffer conditions (from 45.6 to 86 ng mL^–1^), which might be related to a possible hindrance of the antibody–antigen interaction because of the serum matrix. However, the RBD-biofunctionalized surface exhibited a LOD of 21.1 ng mL^–1^, very similar to that obtained under standard buffer conditions. Under these conditions, both assays were further evaluated with real clinical samples.

In order to study the reproducibility of the assays in serum dilution, the interassay variability (replicates within different sensor chips) expressed as CV % (i.e., the ratio of the standard deviation to the mean value in percentage) was studied. The values obtained for the N-protein and RBD domain were below the maximum variability recommended for clinical analysis (15%)^40^ (Table S1), overall confirming a good reproducibility of the assays (i.e., very low variability coming from chip biofunctionalization and/or sample handling and preparation) and thus, the suitability of these viral antigens for polyclonal antibody detection.

### Preliminary Assessment of Clinical Samples

We first evaluated a set of 15 clinical serum samples from 15 different patients (VH.1 collection consisting of 10 COVID-19 positive samples and 5 negative samples, collected in 2016 and stored in the Sepsis Bank of the Vall d’Hebron University Hospital Biobank). Positive serum samples were collected from patients previously diagnosed with COVID-19 by PCR and who had a positive result of specific IgG and IgM class antibodies against the S1 subunit of SARS-CoV-2, as described for the ELISA-IgG-S and ELISA-IgA-S. All the samples were analyzed with N-coated and RBD-coated sensor chips, and a statistical comparison of both N-based and RBD-based serological assays was carried out. As can be seen in Figure 3B, the N-based assays showed poor differentiation between both sample groups, not being statistically significant (*p* > 0.9999). RBD-based assay performed better as the *p*-value (*p* = 0.0208), below 0.05, indicates that the discrimination between positive and negative samples does reach statistical significance.

This result is in concordance with the respective calibration curves (Figure 3A) and the better sensitivity and detectability levels reached with the RBD-based assays. In addition, RBD-based assay shows less dispersion of the negative sample values compared to the N-based assay, resulting in the absence of false negative (or indeterminate) values. Table S1 compares RBD- and N-based assays analytical parameters, where the RBD sensor shows better sensitivity (slope = 0.261 nm mL μg^–1^, *R*^2^ = 0.999, and LOD = 23.9 ng mL^–1^) than the N-sensor (slope = 0.0502 nm mL μg^–1^, *R*^2^ = 0.9016, and LOD = 80.3 ng mL^–1^).

**Figure 4:**
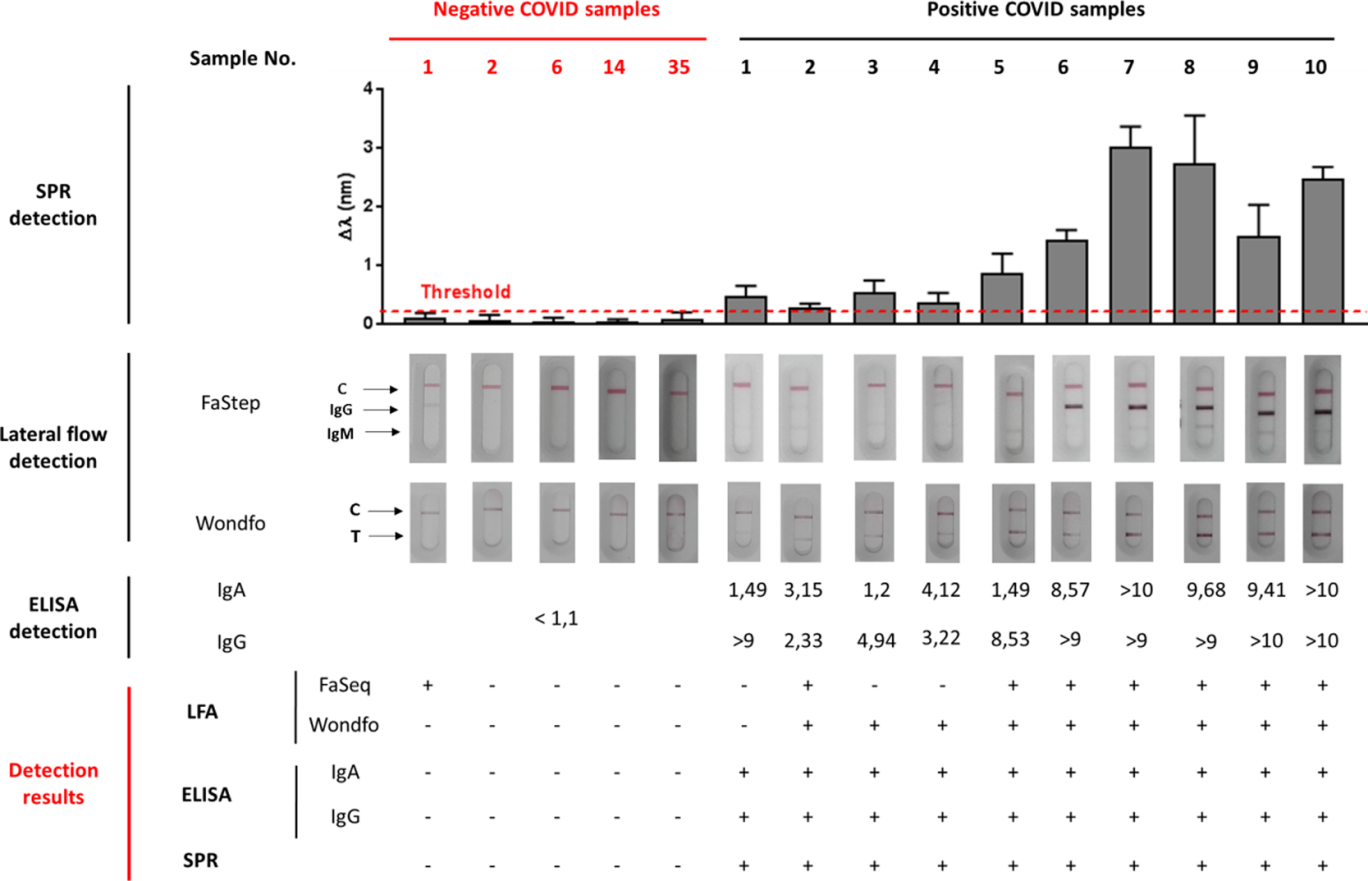
Performance comparison of different COVID-19 serological assays. SPR-biosensor assay, LFA tests, and ELISA tests are shown for positive and negative serum samples. LFA tests were considered as positive after the appearance of a colored band with regular (2) or strong (3) intensity in the IgG and/or IgM line, and negative for very weak (1) or noncolored bands (0). ELISA tests were considered positive for numeric values of IgG and/or IgA cutoff index (COI) > 1.1. SPR biosensor assays were considered positive for samples above the set threshold (red dotted line) calculated, as described in the Experimental section. Detection result rows show the numbers of positive (+) and negative (−) samples for each serological methodology.

To improve the discrimination between negative and positive samples, we assessed the performance of a serological assay employing a mixed sensor chip combining both the RBD and N antigens to capture antibodies targeting both proteins. Figure 3A shows that the multiantigen sensor surface significantly increased the detection signals of a mixture of both pAbs, reaching a better LOD than using the antigens individually. The limit of detection was of 12.75 ng mL^–1^ with a slope of 0.475 nm mL μg^–1^ (*R*^2^ = 0.997), and the calibration curve still shows a broad dynamic range (i.e., at high concentrations such as 10,000 ng mL^–1^), enabling the detection and quantification of antibodies even at high concentrations. Interestingly, we can observe in Figure 3B that the analysis of real samples with the combined serological assay reveals higher responses, derived from the capture of both N and RBD antibodies. Moreover, the negative samples also exhibit lower signals than the single N- and RBD-based assays, reflecting more specificity, which significantly reduces the threshold and its standard deviation (see Figures 3B and 4). From these factors, multianalyte surface is able to discriminate anti-SARS-CoV-2 positive samples from negative samples with the most relevant statistical significance (*p* = 0.0005), notably improving the performance of the individual antigen assays. The reproducibility study, which was performed for viral antigens individually, was also performed for the multianalyte combination of RBD and N antigens (Table S1). As isolated antigen conditions, CV values related to the multianalyte assay were also below 15%. These data support the good reproducibility of the assay and the aptness of multianalyte conditions for the SARS-CoV-2 serological test.

The RBD/N-based serological biosensor assay was qualitatively compared to standard ELISA performed in clinics as well as to two different commercial lateral flow serological tests: Wondfo, which detects the total Igs (against S protein) and FaStep, which detects both IgG and IgM against N and S1 proteins. We employed both LFA to analyze the 15 clinical samples. Results are summarized in Figure 4. The results obtained with ELISA, which detects IgG and IgA antibodies against S1 protein, are also included. As can be observed, the SPR serological assay result precisely concurs to the commercial microplate-based assay, achieving promising sensitivity and specificity. Despite the ELISA not providing quantitative information, a significant correlation between the relative numeric index obtained using this method (COI, cutoff index, extracted from the relative signal of the sample and a control calibrator), and the signal obtained with the SPR assay was observed for most of the samples, which might reflect the good accuracy of the biosensor assay. Interestingly, when analyzing the SPR quantitative detection results for COVID-19-positive samples, the values reveal a clear difference between two groups of samples, 1–5 and 6–10. The first set (1–5) corresponded to patients with mild symptomatology, while samples 6–10 were obtained from ICU-admitted patients. The SPR signals evidence higher levels of Ig for those patients with severe symptomatology compared with the ones with mild conditions. Moreover, LFA tests failed to identify some of those positive samples (i.e., FaStep 1, 3, and 4 and Wondfo 1) and wrongly identified as positive one negative sample (FaStep 1). These LFA results are in concordance with several systematic analysis, which evidence deficient sensitivity and specificity of some LFA assays.^14,15^

Finally, in order to prove the quantification performance of the SPR biosensor and eventually facilitate its comparison with other serology assays detecting the same class of immunoglobulins, we carried out a calibration curve with the first WHO international standard for anti-SARS-CoV-2 immunoglobulin with the concentration expressed in the arbitrary unit of BAU mL^–1^. Calibration curves were generated in the same conditions, as previously described for commercial pAb, in both standard buffer conditions (PBST+DS) and 10% diluted serum. Figure S2 in the Supporting Information shows no differences between the calibration curves depending on conditions, achieving similar limits of detection, 0.098 BAU·mL^–1^ for PBST+DS and 0.137 BAU·mL^–1^ for diluted serum. According to this, patients’ samples were analyzed, and its immunoglobulin concentration was expressed in this standardized units.

### Clinical Validation of SPR-Based COVID-19 Serology

Based on the results achieved with the preliminary clinical evaluation, a larger clinical validation study was initiated. A total of 120 clinical samples were analyzed, including 100 COVID-19-positive clinical samples collected during the pandemic, with confirmed SARS-CoV-2 PCR test and 20 negative samples collected prior the outbreak (Tables 1 and S2). The serum sample collection was carried out between >10 days to months after the PCR results, and they were assessed using the SPR biosensor as well as different commercial techniques such as ELISA, CLIA, and LFA (details on the different tests employed in the experimental section and the Supporting Information).

**Table 1:** Clinical Sample Classification/Characterization

	total	positive^a^	negative	characterization^b^
Vall d’Hebron Hospital (VH)	80	60	20	**VH.1** (*n* = 10)ELISA (10/10)LFA (10/10)
				**VH.2** (*n* = 50)CLIA (50/50)
Clinic Hospital (CH)	40	40	0	**CH.1** (*n* = 40)LFA (40/40)
				mild (*n* = 14)moderate (*n* = 14)severe (*n* = 12)
total	120	100	20	

a Samples from patients with a positive PCR.

b Characterization of positive samples is summarized in Table S2 (Supporting Information).

**Figure 5:**
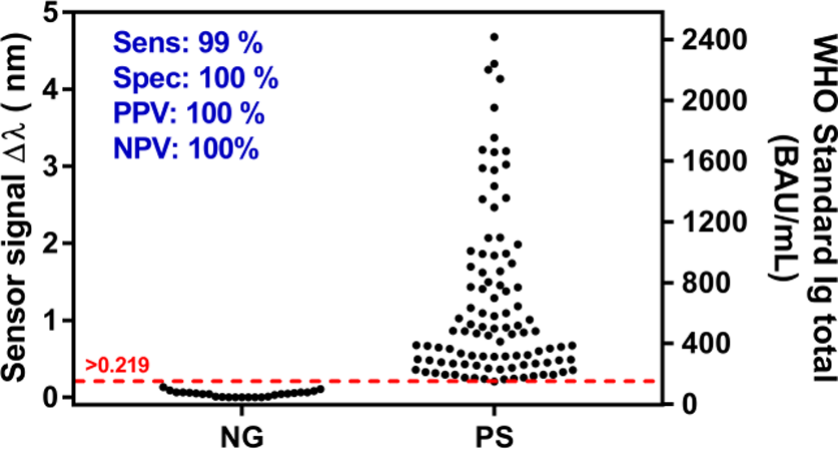
Sensor signal distribution of 100 COVID-19 positive (PS) and 20 negative (NG) clinical samples. Total Ig concentration calculated from the WHO standard calibration curve is shown in the right axis. Sensitivity, specificity, PPV, NPV, and threshold are also shown.

Figure 5 shows the distribution of the sensor signal obtained employing our biosensor assay for each of the samples. Positive samples showed a variable distribution of Ig levels which might go from a few BAU·mL^–1^ to thousands of BAU·mL^–1^. The threshold value was determined from the previous assessment study employing confirmed negative serum samples obtained before the COVID-19 pandemic (Table S3). All the 20 negative samples studied gave signals below the threshold, while only one of the PCR positive samples was considered not positive (indeterminate). The SPR-based serological test shows a sensitivity and PPV of 99 and 100%, respectively. On the other hand, it was able to discriminate negative cases, with a specificity and NPV of 100% both.

To evaluate and validate the accuracy of our SPR serological biosensor, we compared our results with the methods and techniques employed in the two hospitals. Commercial LFAs employed in this study report sensitivities between 90 and 95%. On the other hand, for standard clinical techniques such as CLIA and ELISA, sensitivities usually exceed 95%, reaching 100% in some cases. All cited methodologies have reported specificities between 97 and 99.8%. Table 2 summarizes the diagnostic results obtained for the whole collection of COVID-19 positive samples (VH.1, VH.2, and CH.1) when analyzed by ELISA, CLIA, LFAs, and our SPR biosensor. LFA results, classified according to the intensity scale described in the Experimental section (i.e., 0-no visible color change, 1-weak, 2-regular, and 3-strong), were categorized as negative (intensity 0), indeterminate (intensity 1), and positive (intensity 2 and 3). ELISA, CLIA, and SPR results were categorized depending on the determined threshold for each technique (Table S2 provides all data obtained with each technique).

**Table 2:** Summary of COVID-19 Clinical Samples Validation

VH.1	PCR	SPR	ELISA Euroimmun	LFA Wondfo	LFA FaStep
positive	10	10	10	9	7
indeterminate	0	0	0	0	2
negative	0	0	0	1	1
**VH.2**	**PCR**	**SPR**	**CLIA Liaison**	**CLIA Elecsys**	
positive	50	49	46	48	
indeterminate	0	1	4^a^	0	
Negative	0	0	0	2	
**CH.1**	**PCR**	**SPR**	**LFA Vazyme**	**LFA Quick Profile**	
positive	40	40	18	18	
indeterminate	0	0	4	4	
negative	0	0	18	18	

a ELISA (Euroimmun) was performed to confirm indeterminate results.

In view of the results of Table 2, we can affirm that our SPR biosensor equalizes and even outperforms the different approved diagnostic techniques employed in this study in terms of sensitivity and specificity for the number of samples tested. Considering decentralized technologies, the SPR biosensor can provide highly accurate detection of COVID-19 antibodies in a 15 min assay time (same as LFA), with a diagnostic reliability equivalent to ELISA and CLIA. Therefore, we herein have demonstrated the benefit promised by label-free plasmonic biosensor technology: simple, rapid, and reliable diagnostics.

### Relationship between Humoral Immunity in SARS-CoV-2 Infection and Clinical Severity

To test the capabilities of the SPR biosensor for quantitative assessment of acquired immunity, a preliminary study was carried out to ascertain the existence of a possible correlation between the severity outcome and the levels of SARS-CoV-2 antibodies in sera (as seemingly observed in the preliminary assessment with VH.1 collection). The study was performed with a set of samples after a daily screening in the Clinic Hospital of Barcelona (collection CH.1, *n* = 40). Patients with SARS-CoV-2 antibodies were confirmed by LFA, identifying the presence of IgG and IgM anti-SARS-CoV-2. To stratify patients according to severity and the symptomatology, the date of symptoms onset, symptoms description, hospital or ICU admission, and the length of stay were analyzed. Finally, 40 serum samples from convalescent COVID patients with diverse severity [mild (*n* = 14), moderate (*n* = 14), and severe (*n* = 12)] were included on the validation assay with the plasmonic biosensor. All included patients were symptomatic without statistical difference on symptoms between groups (see the Supporting Information). The time since symptoms onset until sample collection differed between groups (in days) 52.00[44.75–63.25], 76.00 [67.00–88.00], and 118.50[73.50–123.75], *p* < 0.0001 (Figure S3). This was attributed to a more deteriorated health status in severe patients who needed more recovery time from symptoms onset to the inclusion visit.^41^

Although LFA showed limited sensitivity for the detection of anti-SARS-CoV-2 antibodies in some cases, especially in mild severity patients, a correlation was detected between the intensity of the LFA and COVID severity. Related to IgG, 85.7% of moderate and 83.3% of severe patients had positive LFA for IgG (*p* = 000.1), whereas 100% of mild patients had a negative LFA result for IgG. With regard to IgM, 100% of mild and 93% of moderate patients had negative LFA results, but 42% of severe patients had positive LFA for IgM (*p* = 0.007). Both IgG and IgM were present only in severe patients, showing in IgG case a higher intensity of the line LFA result than moderate cases (Table S2). Contrarily, no immunoglobulins were detected in mild patients. Thus, LFA assays showed a possible association between the humoral immunology response and clinical severity because of SARS-CoV-2 infection.

**Figure 6:**
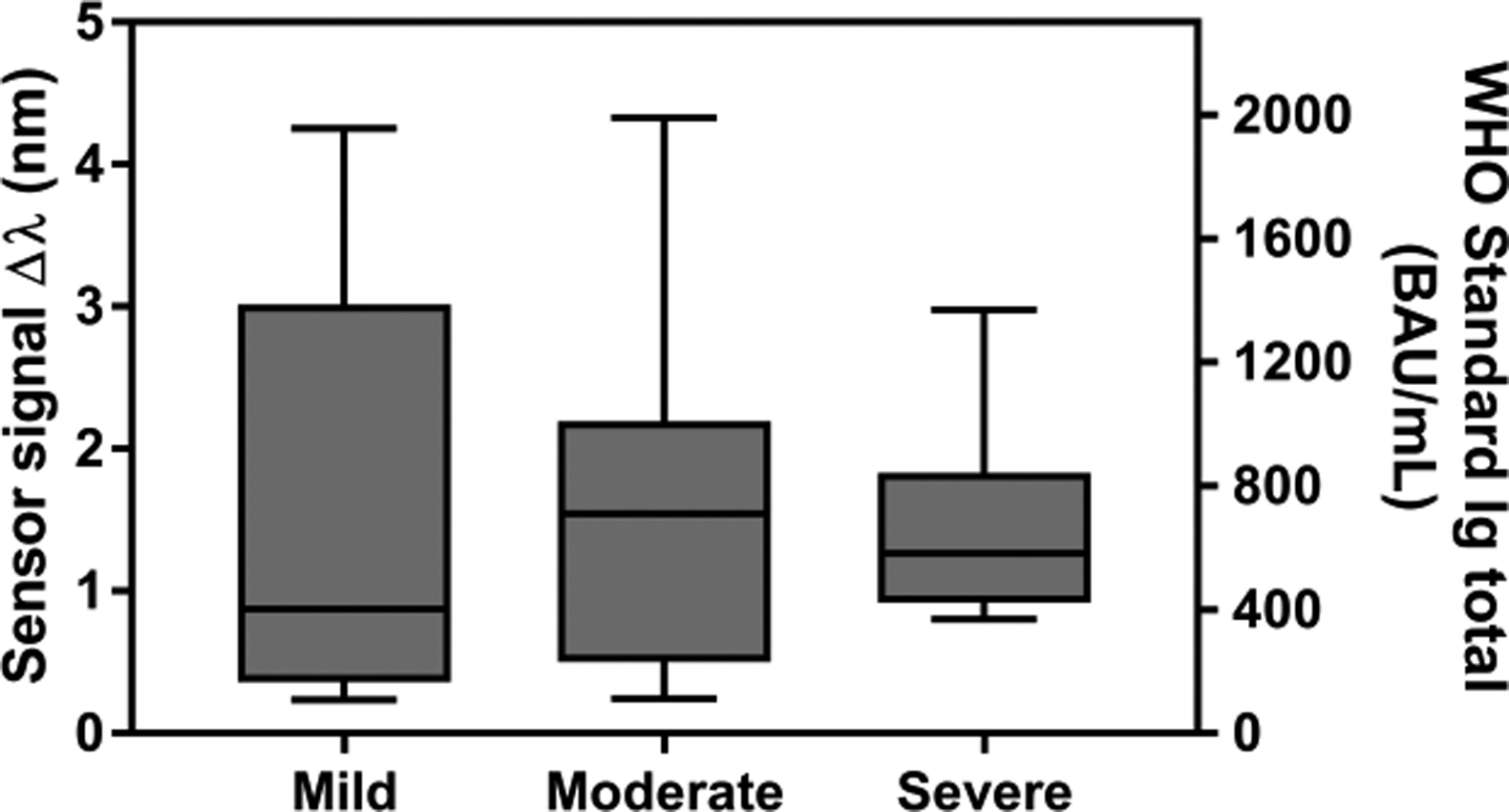
Correlation outcome severity vs antibody concentration. Sensor signal of 40 COVID-19 positive samples from individuals with different degrees of severity (mild, moderate, and severe symptoms). Spearman test (*p* = 0.05). Total Ig concentration calculated from the WHO standard calibration curve is shown in the right axis.

In contrast to LFA, for all patients, regardless of the severity group, we detected immunoglobulin levels with the plasmonic biosensor. The antibody levels were more elevated in the moderate and severe groups versus the mild one. However, the levels of immunoglobulins did not differ statistically between groups (0.87[0.36–3.02], 1.44[0.50–1.83], and 1.07[0.92–1.80]]) *p* = 0.548 (Figure 6). In addition, we did not find the correlation between the levels of antibodies and COVID-19 symptomatology and severity (*r* = 0.175 and *p* = 0.279).

From this preliminary study, and with the limited pool of samples analyzed, we can assert that the SPR biosensor technology shows high sensitivity for identifying total SARS-CoV-2 immunoglobulins, but so far, it has not provided conclusive information regarding a possible correlation between the severity degree and immunity response reflected as SARS-CoV-2 antibody concentration in serum. Although others publications cannot confirm this relationship either,^42−44^ most studies reported in bibliography state that as in the case of Middle East respiratory syndrome (MERS) virus infection, there is a strong association between humoral immunity response and severity outcome after SARS-CoV-2 infection.^45−48^ The study differences related to the number of samples, the time since the onset of symptoms until sample collection, and other factors as the limitations of each study, could hinder the corroboration of this association. Although we acknowledge the necessity of completing the study with an extended number of samples and a longitudinal study, this pilot study exemplifies the convenience a serological quantitative assay may provide to monitor immune response evolution, even for early samples.

## Conclusions

We have demonstrated and fully validated our biosensor technology, based on SPR, for rapid, less than 15 min, identification and quantification of total SARS-CoV-2 antibodies in blood serum. Different strategies were explored depending on the antigen selected to identify SARS-CoV-2 antibodies (N protein and RBD peptide), achieving the most sensitive and specific results when combining RBD and N antigens onto the SPR sensor chip surface. The multianalyte-based biosensor reached excellent limits of detection in serum (low ng mL^–1^ range) that enable direct one-step detection and quantification of SARS-CoV-2 antibodies in COVID-19 patients, providing an excellent discrimination between positive and negative samples (*p* = 0.0005). Moreover, we have implemented the biosensor assay with the first approved anti-SARS-CoV-2 immunoglobulin standard that will allow for further comparison with other serological assays. We have completed an extended clinical validation with COVID-19 positive and negative samples (*n* = 120) that demonstrate a diagnostic sensitivity of 99% and diagnostic specificity of 100% of our biosensor, outperforming current available techniques like immunoassays and rapid tests. We have also conducted a preliminary study of correlation between the humoral immune response and the clinical severity outcome, although a larger cohort would be necessary to generate more conclusive information.

Overall, the results obtained position our biosensor device as an accurate and robust tool for rapid and reliable COVID-19 serology to be employed both at laboratory and eventually in decentralized settings. In addition, the biosensor compact and user-friendly platform design may pave the way to a smooth technological transfer. This work further illustrates the large versatility that SPR biosensors account to be readily adapted to the detection of different types of target biomarkers, thereby becoming a potential alternative tool for rapid diagnostics with great perspectives in clinical practice implementation.

## Supporting Information Available

Description of the biosensor SPR device and details of reagents and buffers, biosensor chip biofunctionalization, data analysis, clinical sample collections, and additional results for the validation of the methodology (PDF)
