# Supplementary material for: Label-Free Plasmonic Biosensor for Rapid, Quantitative, and Highly Sensitive COVID-19 Serology: Implementation and Clinical Validation
Source: Anal Chem. 2021 Dec 31;94(2):975–84. doi: 10.1021/acs.analchem.1c03850 (PMC8751014; doi:10.1021/acs.analchem.1c03850)
Supplement: Supplementary file 1 — ac1c03850_si_001.pdf [file ac1c03850_si_001.pdf]

Supporting Information

Label-free plasmonic biosensor for rapid, quantitative, and highly sensitive COVID-19 serology: implementation and clinical validation

Olalla Calvo-Lozano[1], Miquel Sierra[1], Maria Soler[1*], Maria Carmen Estévez[1*], Luis Chiscano-Camón[2,3], Adolfo Ruiz-Sanmartin[2,3], Juan Carlos Ruiz-Rodriguez[2,3], Ricard Ferrer[2,3], Juan José González-López[4,5,6] Juliana Esperalba[4,5], Candela Fernández-Naval[4,5], Leticia Bueno[7,8,9], Ruben López-Aladid[7,8,9], Antoni Torres[7,8,9], Laia Fernández-Barat[7,8,9], Sarah Attoumani[10], Rémi Charrel[10], Bruno Coutard[10], and Laura M. Lechuga[1]

1. Nanobiosensors and Bioanalytical Applications Group (NanoB2A), Catalan Institute of Nanoscience and Nanotechnology (ICN2), CSIC, CIBER-BBN and BIST, Campus UAB, 08193 Bellaterra, Barcelona, Spain

2. Intensive Care Department, Vall d'Hebron Hospital Universitari, Vall d'Hebron Barcelona Hospital Campus, Barcelona, Passeig Vall d'Hebron 119-129, 08035, Barcelona, Spain

3 Shock, Organ Dysfunction and Resuscitation Research Group, Vall d'Hebron Research Institute (VHIR), Vall d'Hebron Hospital Universitari, Vall d'Hebron Barcelona Hospital Campus, Passeig Vall d'Hebron 119-129, 08035, Barcelona, Spain

4. Clinical Microbiology Department, Vall d'Hebron Hospital Universitari, Vall d'Hebron Barcelona Hospital Campus, Passeig Vall d'Hebron 119-129,08035, Barcelona, Spain

5. Vall d'Hebron Institut de Recerca (VHIR), Vall d'Hebron Barcelona Hospital Campus, Passeig Vall d'Hebron 119-129, 08035 Barcelona, Spain

6. Department of Genetics and Microbiology, Universitat Autònoma de Barcelona, Plaça Cívica, 08193 Bellaterra, Barcelona, Spain

7. Cellex Laboratory, CiberRes (Centro de Investigación Biomédica en Red de Enfermedades Respiratorias, 06/06/0028), Institut d'Investigacions Biomèdiques August Pi i Sunyer (IDIBAPS), Carrer de Roselló 149, 08036 Barcelona, Spain

8. School of Medicine, University of Barcelona, Carrer de Casanova, 143, 08036 Barcelona, Spain

9. Department of Pneumology, Thorax Institute, Hospital Clinic of Barcelona, Carrer de Villarroel, 170, 08036 Barcelona, Spain

10. Unité des Virus Émergents (UVE: Aix-Marseille Univ-IRD 190-Inserm 1207), Marseille, France

** Corresponding authors:* *M.-Carmen Estevez: mcarmen.estevez@icn2.cat*
Maria Soler: maria.soler@icn2.cat

Table of Content

1. Supplementary text
   1.1. Chemical and biological reagents S-3
   1.2. SPR biosensor device S-3
   1.3. Plasmonic sensor chip preparation S-4
   1.4. Clinical samples collection S-4
   1.5. Stratification of convalescent COVID patients. Samples collection from Clinic Hospital (Barcelona) S-5
   1.6. Standard analytical techniques (ELISA, CLIA and LFA) S-5
   1.7. Data analysis S-6
   1.8. Diagnostic sensitivity and specificity S-7
2. Figures S1-S3 S-8
3. Tables S1-S3 S-9
4. References S-15

1.1. Chemical and biological reagents

All the buffer compounds, PBS 50 mM (50 mM phosphate buffer, 0.75 M NaCl, pH 7), MES 0.1 M (2-(N-morpholino) ethanesulfonic acid), HEPES (10 mM 4-(2-hydroxyethyl)piperazine-1-ethanesulfonic acid, 300 mM NaCl, pH 8), Tween 20, dextran sulfate sodium salt (DS) (MW~40000 g mol-1), 16-mercaptohexadecanoic acid (MHDA), (1-ethyl-4(3-dimethylaminopropyl)carbodiimide hydrochloride (EDC), ethanolamine and N-hydroxysulfosuccinimide (s-NHS)) were purchased from Sigma-Aldrich (Steinhem, Germany). Poly-L-lysine-graft-PEG (PLL-g-PEG) was purchased to SuSoS (Dübendorf, Switzerland). Commercial serum was obtained from Sigma–Aldrich (Steinhem, Germany). Recombinant RBD protein and its respective polyclonal IgG antibody (pAb-RBD) were obtained from SinoBiological (Eschborn, Germany). A polyclonal IgG antibody specific for N protein (pAb-N) was purchased from GeneTex (Irvine, CA, US). WHO International Standard Anti-SARS-CoV-2 immunoglobulin (code: 20/136) obtained from pooled plasma from individuals recovered from SARS-CoV-2 infection, was purchased from National Institute for Biological Standards and Control (NIBSC, Hertfordshire, United Kingdom). Monoclonal IgG antibody against CRP C7 (anti-CRP) was acquired from HyTest (Turku, Finland). The coding sequences of the N and C-terminal domains (positions 1-180 aa and 247-418 aa, respectively) of the SARS-CoV-2 N protein (Accession No. MT777677) were cloned in a pET 28a plasmid in fusion with a hexahistdine tag coding sequence at the 3'end. Recombinant proteins were produced in E. coli T7 Express (DE3) cells (New England Biolabs) and purified under non-denaturing conditions by Immobilised Metal Affinity Chromatography followed by Size Exclusion Chromatography. Proteins were stored in 10 mM HEPES, 150 mM NaCl, pH 7.5 buffer. N and C-terminal domains were mixed in mass ratio 1/1.

1.2. SPR biosensor device

The customized SPR employed in this work (**Figure S1A**) is based on the Kretschmann configuration and works at a fixed angle of incidence (θ=70°). The SPR biosensor monitors the binding events in real-time by tracking the SPR-wavelength displacements (Δλ, nm). Plasmonic sensor chips are clamped between a trapezoidal glass prism through RI matching oil (n≈1.512) and a custom-made flow cell. The flow cell is connected to a microfluidics system consisting of a syringe pump with adjustable pumping speed that provides a constant liquid flow and a manually operated injection valve incorporating a 100 µL loop to deliver the liquid samples into the microfluidic cell. The sensor surface is excited by a collimated halogen light source set in TM polarization. The polarized light reaches the substrate through the prism coupling, generating an evanescent field at the sensor surface which is very sensitive to refractive index changes (RI). The reflected light is collected and fiber-coupled to a CCD spectrometer.

Biomolecular interactions occurring at the gold sensor surface result in an increment in the mass, which translates in an increase in the RI (shifting the resonance curve to higher wavelengths), whereas desorptions from the surface decrease the RI, shifting the curve to lower wavelengths. The tracking of the resonance peak position (Δλ) can be followed in real time via polynomial fit using a custom-made readout software, being possible to detect interactions or desorptions in real-time (**Figure S1B**).

1.3. Plasmonic sensor chip preparation

Gold sensor chips were fabricated by metal evaporation employing an electron beam-deposition system (AJA International Inc. ATC-8E, Orion, USA) (1 nm of titanium and 49 nm of gold). Prior to surface biofunctionalization a cleaning procedure was followed. Firstly, plasmonic sensor chips were cleaned by consecutive heating at 80ºC and sonicating 1 min with solvents of increasing polarity (i.e. acetone, ethanol and MilliQ water). Then, sensor chips were dried with a N_2 flow and placed in a UV/Ozone Procleaner Plus (Bioforce Nanosciences, Utah, US) for 20 min for surface activation. The sensor chips were finally rinsed with ethanol and dried with N_2 flow. Then, the sensor chips were chemically modified through the formation of a self-assembled monolayer (SAM) of carboxyl groups with 16-mercapto-hexadecanoic acid (MHDA). The chips were rinsed with ethanol and dried with N_2 stream and placed on the SPR instrument for in-situ covalent immobilization of the viral proteins (N protein, RBD peptide or multianalyte (N+RBD. 1:1)) to carboxyl groups on the gold sensor chip through EDC/s-NHS chemistry[1,2]. For all the immobilized surfaces, a final blocking step was included to avoid non-specific adsorptions employing PLL-g-PEG [3,4]. Finally, the sensor chips were kept under a continuous flow of PBST+DS (PBS 10 mM + 0.5% Tween 20 + 2 mg mL^{-1} DS) at 15 µL min−1. Figure S1B shows a representative antigen immobilization of the three-step reaction involved in the covalent coupling to the gold sensor chip.

1.4. Clinical samples collection

A total of 125 clinical samples were collected from two hospitals in Barcelona (Spain) in three different batches. Two batches were provided by Vall d'Hebron University Hospital (VH.1 n=15, and VH.2 n=70), and a third batch was provided by the Clinic Hospital of Barcelona (CH.1 n=40). VH.1 and VH.2 serum samples and data from the patients used in this study were provided by the Clinical Microbiology Department and by the Sepsis Bank of Vall d'Hebron University Hospital Biobank (PT17/0015/0047), integrated in the Spanish National Biobanks Network. Samples were processed following standard operating procedures with the appropriate approval of the Clinical Research Ethics Committee (approval reference numbers for Vall d'Hebron University Hospital PR(AG)11/2016 and PR(AG297/2020)). VH.1 samples consisted of 15 serum samples from 15 different patients (5 negative pre-pandemic samples collected in

2016, and 10 COVID-19 positive samples, among which 5 were from patients with mild symptoms (i.e paucisymptomatic) and 5 from patients with severe symp-toms (i.e. patients admitted to the ICU). VH.2 samples included 20 pre-pandemic negative samples and 50 COVID-19 positive samples, confirmed by PCR, ELISA and CLIA. No information about VH.2 batch symptoms severity was collected due to the pressing urgency of the pandemic in its first wave.

CH.1 serum samples included 40 COVID-19 positive samples that were collected by the Clinic Hospital of Barcelona following a symptomatology study. Samples included adult patients (>18 years old) with SARS-CoV-2 symptomatology and with confirmed positive diagnosis during symptoms and confirmed negative diagnosis at infection resolution by PCR of nasopharyngeal swab, tracheal aspirate, or bronchoalveolar aspirate. Patients with HIV, hepatitis and immunosuppressed patients were excluded. Convalescent COVID-19 patients were cited to perform a rapid questionnaire of symptoms and confirm by LFA the presence of anti-SARS-CoV-2 immunoglobulins. After this confirmation, a blood sample was obtained, and serum was processed and stored at a local biobank at -80ºC until analysis. Date of symptoms onset, symptoms description, Hospital or ICU admission and length of stay was ana-lyzed in order to stratify patients according to severity: mild (symptomatic with no hospitalization), moderate (re-quired hospital admission), and severe (required ICU admission) and symptomatology. The study was carried out in compliance with the Declaration of Helsinki (current version, Fortaleza, Brazil, October 2013) and was conducted in accordance with the requirements of the 2007 Spanish Biomedical Research Act. The study was approved by the institution's Internal Review Board (registry number HCB/2020/0332). Written informed consent was obtained from all patients.

1.5. Stratification of convalescent COVID patients. Samples collection from Clinic Hospital (Barcelona)

The stratification of COVID patients was performed according to the severity and symptomatology date of symptoms onset, symptoms description, Hospital or ICU admission and length of stay. All included patients were symptomatic without statistical difference on symptoms between groups (see figure S3). In brief, the frequency of each symptom by mild, moderate or severe was respectively: fever (71%, 79% and 100%, p=1.43), myalgias (57%, 14% and 33 %, p=0.059), cough (57%, 57%, 67%, p=0.853), anosmia (50%, 36%, 42%, p=0.745), ageusia (36%, 21%, 33%, p=0.680), diarrhea (21%, 36%, 50%, p=0.313), headache (21%, 39%, 33%, p=0.790), dyspnea (21%, 36%, 58%, p=0.151).

1.6. Standard analytical techniques (ELISA, CLIA and LFA)

Serological response from the samples provided by Vall d'Hebron University Hospital (VH.1 and VH.2) was determined by ELISA and CLIA commercial kits. ELISA-IgG-S and ELISA-

IgA-S kits, which detect IgG and IgA antibodies against spike SARS-CoV-2 glycoprotein (Anti-SARS-CoV-2 ELISA (IgG) (EUROIMMUN, Lübeck, Germany) were performed on the EUROIMMUN Analyzer I-2P (EUROIMMUN, Germany). ELISA tests have proven 96.9% and 94.4% sensitivity (SE) and 98.3 % and 99.6 % specificity (SP) for IgA and IgG, respectively. Samples resulting in cut-off index (COI) ≥1.1 were considered interpreted as positive. Besides, two different CLIA commercial kits were employed: (CLIA-1) CLIA-IgG-S kit: Liaison SARS-CoV-2 S1/S2 IgG (DiaSorin, Italy), performed on the LIAISON® XL Analyzer (DiaSorin, Italy) for quantitative determination of the spike (S) glycoprotein subunits 1 and 2 (S1/S2) with 97.4 % (SE) and 98.5 % (SP). The result was considered positive when the COI ≥ 15 AU/mL; (CLIA-2) ECLIA-Igs-N kit: Elecsys® Anti-SARS-CoV-2 (Roche Diagnostics, USA), performed on the Cobas 8800 system (Roche Diagnostics, USA) for qualitative determination of total antibodies (including IgG, IgM and IgA) against N SARS-CoV-2 protein with 100 % (SE) and 99.80 % (SP). The result was considered positive when COI ≥ 1.0.

Lateral flow assays (LFA) were acquired from different suppliers: (LFA-1) 2019-nCoV IgG/IgM Detection Kit (Colloidal Gold-Based) Vazyme (Vazymebiotech Co, Nanjing, China) with 91.54% (SE) and 97.02% (SP); (LFA-2) Quick Profile 2019-nCoV IgG/IgM Test Card (Quick Profile, USA) with 87,8% (SE) and 99% (SP); (LFA-3) Wondfo Biotech (Guangzhou, Luogang, China) with 86.43% (SE) and 99.57% (SP); and (LFA-4) FaStep (Hangzhou, China) that identify IgM and IgG with 93.7% (SE) and 99.1% (SP). LFA tests were performed following the manufacturer’s instructions. Briefly, a drop of blood or 10 µL of serum and 2 drops of buffer solution were added to the corresponding loading area in the cassettes. After 15 min, the result was reflected by the appearance of colored bands. The LFA result was qualified based on the intensity of the Ig bands as follows: strong (3), regular (2) weak (1), and (0) no color change observed.

1.7. Data analysis

The real-time sensorgrams were processed extracting the final response (Δλ) after signal stabilization once the whole sample volume has passed through the flow cell. For the flow rate employed and the sample volume, this corresponds to approximately 1000 s after injection. The biosensor data were analyzed and processed using Origin 8.0 software (OriginLab Northampton, MA). Data and statistical analysis (one way ANOVA test) were performed using Graphpad Prism (Graphpad Software, Inc., California, US). Calibration curves were obtained by evaluating different concentrations of the polyclonal antibodies in triplicate. The mean sensor signal (Δλ) and its standard deviation (SD) were plotted *versus* polyclonal antibody concentration. The data was fitted to a lineal regression equation (y=mX+b) where y is the sensor response, X is the concentration of polyclonal antibody, m is the slope of the linear regression curve and b is the intercept. The limit of detection (LOD) for each antibody was

calculated as the concentration corresponding to a blank signal plus three times its standard deviation. The coefficients of variation were obtained as the ratio of the standard deviation of the mean, expressed in percentages (% CV).

The differences between groups were analyzed with Kruskal-Wallis test, considering a p value $p < 0.05$ to be statistically significant. Correlation between immune response and clinical severity was analyzed by Spearman test considering p value < 0.05. Threshold values (cut-off values) to determine positive samples were calculated from the mean + 2SD of control negative samples. A value < 0.9xMean +2SD was considered negative; a value > 1.1xMean + 2SD as positive; and between 0.9 - 1.1xMean + 2SD as indeterminate. The diagnostic sensitivity (SE), diagnostic specificity (SP), positive predictive value (PPV) and negative predictive value (NPV) were determined as described in the SI.

1.8. Diagnostic sensitivity and specificity

Sensitivity (SE), specificity (SP), positive predictive value (PPV) and negative predictive value (NPV) were obtained from the number of false positives (FP), false negatives (FN), true positives (TP) and true negatives (TN).

	**PCR Positive**	**PCR Negative**
**SPR Test Positive**	TP	FP
**SPR Test Negative**	FN	TN

$$SE = \frac{\mathrm{TP}}{\mathrm{TP} + \mathrm{FN}} \quad SP = \frac{\mathrm{TN}}{\mathrm{TN} + \mathrm{FP}}$$

$$PPV = \frac{\mathrm{TP}}{\mathrm{FP} + \mathrm{TP}} \quad NPV = \frac{\mathrm{TN}}{\mathrm{FN} + \mathrm{TN}}$$

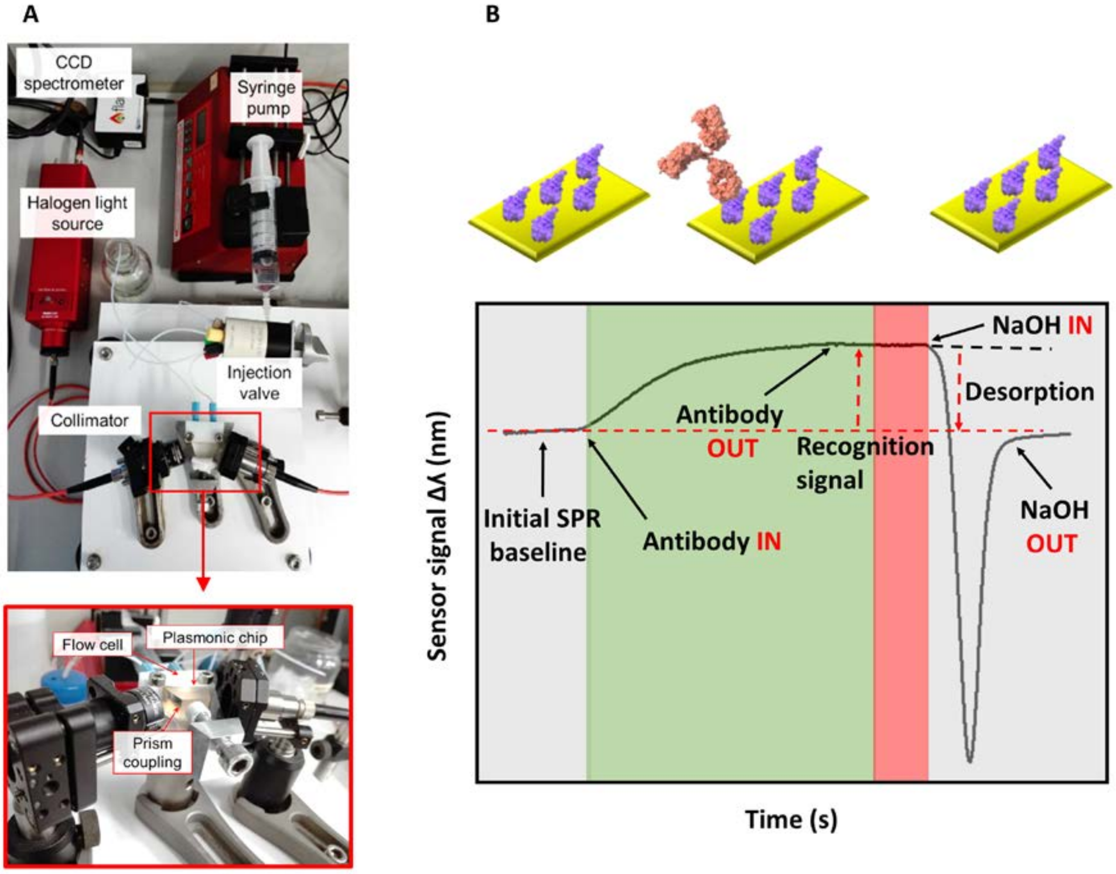

Figure S1. SPR device. A) Photograph of the experimental SPR set-up. **B)** Real-time monitoring of the wavelength displacement (Δλ) depending on refractive index changes onto the sensor surface. Increase in the sensor signal can be observed when a recognition event antigen-antibody occurs. Desorption event generated by regeneration step is represented by the decrease of the sensor signal and return to the baseline.

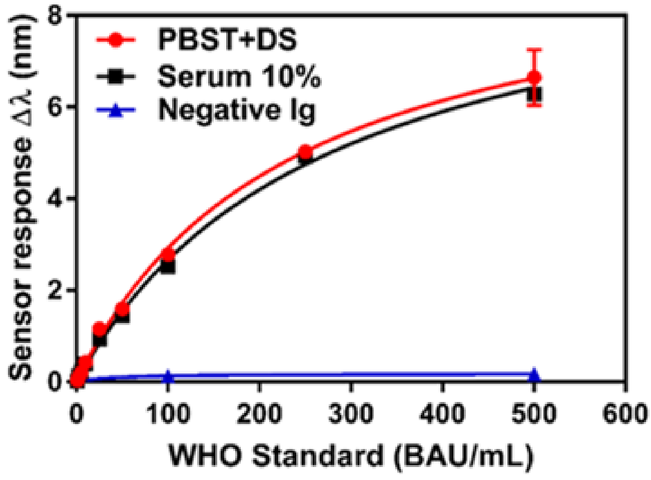

Figure S2. Calibration curves obtained using the WHO Standard anti-SARS-CoV-2 immunoglobulin in PBST+DS and 10% diluted commercial serum using RBD+N biofunctionalized sensor surface. Biosensor response represents the mean ± SD of three evaluations.

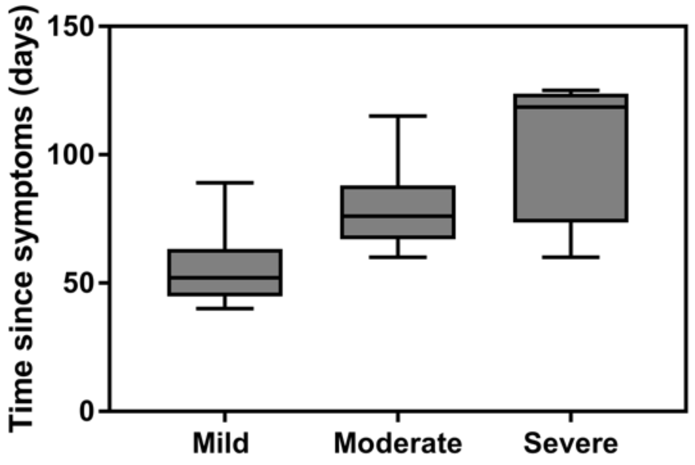

Figure S3. **Recovery time from symptoms onset to the inclusion visit.** Time since initiation of symptoms until sample obtaining depending on the clinical severity degree (collection CH.1, n=40).

Table S1. Inter-assay variability for diluted serum (10%) calibration curves (CC)

Viral antigen	Parameter	CC1	CC2	CC3	Mean±SD	CV (%)
**N-sensor**	**Slope ($x10^{-5}$ nm·mL·ng^{-1})**	5.7	4.8	4.65	5.02 ± 0.6	**11.1**
	**LOD (ng·mL^{-1})**	70.7	84.2	86	80.3 ± 8.4	**10.4**
**RBD-sensor**	**Slope ($x10^{-5}$ nm·mL·ng^{-1})**	22.5	27.1	28.7	26.1±3.2	**12.3**
	**LOD (ng·mL^{-1})**	26.7	22.1	23	23.9 ± 2.4	**10.1**
**Multianalyte N+RBD-sensor**	**Slope ($x10^{-5}$ nm·mL·ng^{-1})**	54.1	42.9	45.5	47.5±5.9	**12.3**
	**LOD (ng·mL^{-1})**	11.1	14	13.2	12.7 ± 1.5	**11.7**

Table S2. Techniques employed for patient's serum samples characterization*

Technique	*ELISA*[1]		*CLIA*[2]		*Rapid tests (LFA)*[3]							*SPR*[4]	
Brand	***Euroimmun***		***1-Liaison***	***2-Elecsys***	***1-Vazyme***		***2-Quick Profile***		***3-Wondfo***	***4-FaStep***		***Serological test***	
Sample	***IgA***	***IgG***	***Ig G***	***Ig Total***	***IgG***	***IgM***	***IgG***	***IgM***	***Total Ig***	***IgG***	***Ig M***	***Ig total (BAU/mL)***	***SPR (nm)***
VH.1.N1	--	--	--	--	--	--	--	--	0	2	0	17.6	0.063
VH.1.N2	--	--	--	--	--	--	--	--	0	0	0	17.3	0.062
VH.1.N3	--	--	--	--	--	--	--	--	0	0	0	11.9	0.043
VH.1.N4	--	--	--	--	--	--	--	--	0	0	0	17.2	0.062
VH.1.N5	--	--	--	--	--	--	--	--	0	0	0	22.6	0.081
VH.1.P1	1.49	>9	--	--	--	--	--	--	1	0	0	159.5	0.548
VH.1.P2	3.15	2.33	--	--	--	--	--	--	2	1	2	92.9	0.326
VH.1.P3	1.20	4.94	--	--	--	--	--	--	2	0	1	156.6	0.538
VH.1.P4	4.12	3.22	--	--	--	--	--	--	2	1	1	125.9	0.437
VH.1.P5	1.49	8.53	--	--	--	--	--	--	2	1	2	260.3	0.864
VH.1.P6	8.57	>9	--	--	--	--	--	--	2	3	1	458.6	1.427
VH.1.P7	>10	>9	--	--	--	--	--	--	3	3	1	1297.1	3.196
VH.1.P8	9.68	>9	--	--	--	--	--	--	3	3	2	1042.0	2.741
VH.1.P9	9.41	>10	--	--	--	--	--	--	3	3	2	483.8	1.494
VH.1.P10	10	>10	--	--	--	--	--	--	3	3	2	963.1	2.588
VH.2.N1	--	--	--	--	--	--	--	--	--	--	--	29.9	0.107
VH.2.N2	--	--	--	--	--	--	--	--	--	--	--	0.2	0.001
VH.2.N3	--	--	--	--	--	--	--	--	--	--	--	7.7	0.028
VH.2.N4	--	--	--	--	--	--	--	--	--	--	--	0.5	0.002
VH.2.N5	--	--	--	--	--	--	--	--	--	--	--	0.0	0.000
VH.2.N6	--	--	--	--	--	--	--	--	--	--	--	36.7	0.131
VH.2.N7	--	--	--	--	--	--	--	--	--	--	--	17.2	0.062
VH.2.N8	--	--	--	--	--	--	--	--	--	--	--	0.0	0.000
VH.2.N9	--	--	--	--	--	--	--	--	--	--	--	2.7	0.010
VH.2.N10	--	--	--	--	--	--	--	--	--	--	--	0.41	0.001
VH.2.N11	--	--	--	--	--	--	--	--	--	--	--	0	0

Technique	ELISA[1]		CLIA[2]		Rapid tests (LFA)[3]							SPR[4]	
Brand	Euroimmun		1-Liaison	2-Elecsys	1-Vazyme		2-Quick Profile		3-Wondfo	4-FaStep		Serological test	
Sample	IgA	IgG	Ig G	Ig Total	IgG	IgM	IgG	IgM	Total Ig	IgG	Ig M	Ig total (BAU/mL)	SPR (nm)
VH.2.N12	--	--	--	--	--	--	--	--	--	--	--	13.8	0.050
VH.2.N13	--	--	--	--	--	--	--	--	--	--	--	2.3	0.008
VH.2.N14	--	--	--	--	--	--	--	--	--	--	--	13.1	0.047
VH.2.N15	--	--	--	--	--	--	--	--	--	--	--	12.2	0.044
VH.2.N16	--	--	--	--	--	--	--	--	--	--	--	14.9	0.054
VH.2.N17	--	--	--	--	--	--	--	--	--	--	--	16.8	0.060
VH.2.N18	--	--	--	--	--	--	--	--	--	--	--	24.4	0.088
VH.2.N19	--	--	--	--	--	--	--	--	--	--	--	0.0	0.0
VH.2.N20	--	--	--	--	--	--	--	--	--	--	--	12.0	0.043
VH.2.P1	--	2.6**	14.7	7.24	--	--	--	--	--	--	--	248.8	0.829
VH.2.P2	--	1.7**	10.8	0.45	--	--	--	--	--	--	--	190.5	0.647
VH.2.P3	--	1.7**	12.4	4.89	--	--	--	--	--	--	--	197.0	0.668
VH.2.P4	--	--	33.2	5.8	--	--	--	--	--	--	--	154.0	0.53
VH.2.P5	--	1.5**	11.5	6.98	--	--	--	--	--	--	--	107.33	0.375
VH.2.P6	--	--	27.4	0.49	--	--	--	--	--	--	--	129.8	0.450
VH.2.P7	--	--	36.6	66	--	--	--	--	--	--	--	139.0	0.481
VH.2.P8	--	--	59.4	3.39	--	--	--	--	--	--	--	2435.1	4.678
VH.2.P9	--	--	39.6	42.7	--	--	--	--	--	--	--	88.9	0.313
VH.2.P10	--	--	83.1	30	--	--	--	--	--	--	--	143.2	0.494
VH.2.P11	--	--	16.7	2.58	--	--	--	--	--	--	--	83.6	0.295
VH.2.P12	--	--	163	89.7	--	--	--	--	--	--	--	632.8	1.867
VH.2.P13	--	--	18.3	48	--	--	--	--	--	--	--	337.9	1.093
VH.2.P14	--	--	110	125	--	--	--	--	--	--	--	258.3	0.858
VH.2.P15	--	--	46.5	120	--	--	--	--	--	--	--	82.4	0.291
VH.2.P16	--	--	20.4	39.1	--	--	--	--	--	--	--	185.4	0.631
VH.2.P17	--	--	15.4	5.56	--	--	--	--	--	--	--	57.8	0.205
VH.2.P18	--	--	162	49.1	--	--	--	--	--	--	--	1194.8	3.021
VH.2.P19	--	--	31.5	2.23	--	--	--	--	--	--	--	253.4	0.843

Technique	*ELISA*[1]		*CLIA*[2]		*Rapid tests (LFA)*[3]							*SPR*[4]	
Brand	***Euroimmun***		***1-Liaison***	***2-Elecsys***	***1-Vazyme***		***2-Quick Profile***		***3-Wondfo***	***4-FaStep***		***Serological test***	
Sample	***IgA***	***IgG***	***Ig G***	***Ig Total***	***IgG***	***IgM***	***IgG***	***IgM***	***Total Ig***	***IgG***	***Ig M***	***Ig total (BAU/mL)***	***SPR (nm)***
VH.2.P20	--	--	42.6	29	--	--	--	--	--	--	--	120.8	0.420
VH.2.P21	--	--	32.6	20.4	--	--	--	--	--	--	--	361.7	1.161
VH.2.P22	--	--	91.4	156	--	--	--	--	--	--	--	175.5	0.599
VH.2.P23	--	--	87.7	48.8	--	--	--	--	--	--	--	153.7	0.529
VH.2.P24	--	--	94	129	--	--	--	--	--	--	--	100.8	0.353
VH.2.P25	--	--	40.5	4.96	--	--	--	--	--	--	--	139.2	0.481
VH.2.P26	--	--	34.9	2.76	--	--	--	--	--	--	--	118.4	0.412
VH.2.P27	--	--	40.2	79.5	--	--	--	--	--	--	--	71.7	0.254
VH.2.P28	--	--	26.5	20.4	--	--	--	--	--	--	--	193.2	0.656
VH.2.P29	--	--	64.1	89.9	--	--	--	--	--	--	--	67.4	0.239
VH.2.P30	--	--	32.5	12.9	--	--	--	--	--	--	--	245.9	0.820
VH.2.P31	--	--	58.6	78.7	--	--	--	--	--	--	--	288.3	0.948
VH.2.P32	--	--	83.7	80.4	--	--	--	--	--	--	--	165.7	0.568
VH.2.P33	--	--	37.5	134	--	--	--	--	--	--	--	70.5	0.250
VH.2.P34	--	--	17.3	6.4	--	--	--	--	--	--	--	185.2	0.630
VH.2.P35	--	--	366	40.8	--	--	--	--	--	--	--	646.4	1.900
VH.2.P36	--	--	65.4	126	--	--	--	--	--	--	--	157.4	0.541
VH.2.P37	--	--	29.8	3.35	--	--	--	--	--	--	--	141.5	0.489
VH.2.P38	--	--	17.7	36.5	--	--	--	--	--	--	--	681.5	1.982
VH.2.P39	--	--	76.6	43.7	--	--	--	--	--	--	--	324.9	1.055
VH.2.P40	--	--	30.6	83.1	--	--	--	--	--	--	--	82.6	0.291
VH.2.P41	--	--	29.8	18.4	--	--	--	--	--	--	--	71.4	0.253
VH.2.P42	--	--	70.9	11.7	--	--	--	--	--	--	--	439.8	1.377
VH.2.P43	--	--	107	99.3	--	--	--	--	--	--	--	198.1	0.671
VH.2.P44	--	--	18	1.01	--	--	--	--	--	--	--	93.0	0.327
VH.2.P45	--	--	106	1.88	--	--	--	--	--	--	--	1666.4	3.761
VH.2.P46	--	--	22.7	12.8	--	--	--	--	--	--	--	369.3	1.182
VH.2.P47	--	--	20.2	42.2	--	--	--	--	--	--	--	131.3	0.455

Technique	*ELISA*[1]		*CLIA*[2]		*Rapid tests (LFA)*[3]							*SPR*[4]	
Brand	*Euroimmun*		*1-Liaison*	*2-Elecsys*	*1-Vazyme*		*2-Quick Profile*		*3-Wondfo*	*4-FaStep*		*Serological test*	
Sample	*IgA*	*IgG*	*Ig G*	*Ig Total*	*IgG*	*IgM*	*IgG*	*IgM*	*Total Ig*	*IgG*	*Ig M*	*Ig total (BAU/mL)*	*SPR (nm)*
VH.2.P48	--	--	101	40.5	--	--	--	--	--	--	--	1950.9	4.136
VH.2.P49	--	--	29.1	21.6	--	--	--	--	--	--	--	901.2	2.463
VH.2.P50	--	--	25.2	31.2	--	--	--	--	--	--	--	450.2	1.405
CH.1.P1	--	--	--	--	0	0	0	0	--	--	--	109.3	0.382
CH.1.P2	--	--	--	--	0	0	0	0	--	--	--	269.6	0.892
CH.1.P3	--	--	--	--	0	0	0	0	--	--	--	1307.6	3.214
CH.1.P4	--	--	--	--	0	0	0	0	--	--	--	125.7	0.437
CH.1.P5	--	--	--	--	0	0	0	0	--	--	--	65.9	0.234
CH.1.P6	--	--	--	--	0	0	0	0	--	--	--	1404.4	3.371
CH.1.P7	--	--	--	--	0	0	0	0	--	--	--	101.9	0.357
CH.1.P8	--	--	--	--	0	0	0	0	--	--	--	2046.6	4.252
CH.1.P9	--	--	--	--	0	0	0	0	--	--	--	258.0	0.857
CH.1.P10	--	--	--	--	0	0	0	0	--	--	--	103.6	0.363
CH.1.P11	--	--	--	--	0	0	0	0	--	--	--	1154.2	2.949
CH.1.P12	--	--	--	--	0	0	0	0	--	--	--	308.9	1.009
CH.1.P13	--	--	--	--	0	0	0	0	--	--	--	406.4	1.286
CH.1.P14	--	--	--	--	0	0	0	0	--	--	--	82.7	0.292
CH.1.P15	--	--	--	--	0	0	0	0	--	--	--	68.2	0.242
CH.1.P16	--	--	--	--	1	0	1	0	--	--	--	563.1	1.697
CH.1.P17	--	--	--	--	2	0	2	0	--	--	--	539.3	1.637
CH.1.P18	--	--	--	--	0	0	0	0	--	--	--	153.1	0.527
CH.1.P19	--	--	--	--	2	0	2	0	--	--	--	77.6	0.274
CH.1.P20	--	--	--	--	3	0	3	0	--	--	--	1289.7	3.184
CH.1.P21	--	--	--	--	3	0	3	0	--	--	--	122.7	0.427
CH.1.P22	--	--	--	--	2	0	2	0	--	--	--	719.5	2.070
CH.1.P23	--	--	--	--	1	0	1	0	--	--	--	620.9	1.839
CH.1.P24	--	--	--	--	3	0	3	0	--	--	--	214.8	0.724
CH.1.P25	--	--	--	--	3	0	3	0	--	--	--	2112.4	4.329

Technique	*ELISA[1]*		*CLIA[2]*		*Rapid tests (LFA)[3]*							*SPR[4]*	
Brand	*Euroimmun*		*1-Liaison*	*2-Elecsys*	*1-Vazyme*		*2-Quick Profile*		*3-Wondfo*	*4-FaStep*		*Serological test*	
Sample	*IgA*	*IgG*	*Ig G*	*Ig Total*	*IgG*	*IgM*	*IgG*	*IgM*	*Total Ig*	*IgG*	*Ig M*	*Ig total (BAU/mL)*	*SPR (nm)*
CH.1.P26	--	--	--	--	3	0	3	0	--	--	--	468.2	1.453
CH.1.P27	--	--	--	--	1	0	1	0	--	--	--	953.5	2.569
CH.1.P28	--	--	--	--	3	1	3	1	--	--	--	199.9	0.677
CH.1.P29	--	--	--	--	0	0	0	0	--	--	--	276.6	0.913
CH.1.P30	--	--	--	--	1	0	1	0	--	--	--	1169.3	2.976
CH.1.P31	--	--	--	--	3	1	3	1	--	--	--	461.1	1.434
CH.1.P32	--	--	--	--	0	0	0	0	--	--	--	580.4	1.740
CH.1.P33	--	--	--	--	3	1	3	1	--	--	--	720.8	2.073
CH.1.P34	--	--	--	--	3	0	3	0	--	--	--	274.6	0.907
CH.1.P35	--	--	--	--	3	0	3	0	--	--	--	239.7	0.801
CH.1.P36	--	--	--	--	3	1	3	1	--	--	--	282.3	0.930
CH.1.P37	--	--	--	--	3	1	3	1	--	--	--	314.4	1.025
CH.1.P38	--	--	--	--	3	0	3	0	--	--	--	532.9	1.621
CH.1.P39	--	--	--	--	3	1	3	1	--	--	--	630.5	1.862
CH.1.P40	--	--	--	--	3	0	3	0	--	--	--	337.5	1.092

(*) COVID-19 positive clinical samples were analyzed in their corresponding Hospital laboratories during the first and second pandemic waves in Spain (March – September 2020). The different analytical techniques employed for sample analysis correspond to standard regulatory-approved commercial kits available at each collection time.

(**) Samples analyzed by ELISA to confirm CLIA indeterminate results.

(1) ELISA: Euroimmun Test. Values indicated correspond to the cut-off index (COI). Samples with COI ≥1.1 were considered -positive

(2) CLIA: Chemiluminescence immunoassay. CLIA- 1-Liaison: Semiquantitative information Values shown correspond to COI expressed in AU/mL. Samples with COI ≥15 AU/mL were considered positive; CLIA-2-Elecsys: qualitative information. Values shown correspond to COI index. Samples with COI≥1.0 were considered positive.

(3) Rapid diagnostic tests based on lateral flow immunoassays (LFA). Intensity scale of the Ig bands: (0): negative, not colored or visible at naked eye; (1) weak; (2) regular/medium; (3) strong/high intensity.

(4) SPR. Samples were considered positive for values above the set threshold (0.219 nm). Quantitative information was obtained by employing the WHO Standard calibration curve for anti-SARS-COV-2 immunoglobulins.

Table S3. Threshold calculation from negative sample signals

TS calculation (Mean + 2SD)	Result
< **0.179**	Negative
**0.179 – 0.219**	Indeterminate
> **0.219**	Positive

References

[1] Peláez, E. C.; Estevez, M. C.; Mongui, A.; Menéndez, M. C.; Toro, C.; Herrera-Sandoval, O. L.; Robledo, J.; García, M. J.; Portillo, P. Del; Lechuga, L. M. ACS Infect. Dis. 2020, 6, 1110–1120.

[2] Peláez, E. C.; Estevez, M. C.; Domínguez, R.; Sousa, C.; Cebolla, A.; Lechuga, L. M. Anal. Bioanal. Chem. 2020, 412, 6407–6417.

[3] Soler, M.; Estevez, M. C.; Villar-Vazquez, R.; Casal, J. I.; Lechuga, L. M. Anal. Chim. Acta 2016, 930, 31–38.

[4] Peláez, E. C.; Estevez, M. C.; Portela, A.; Salvador, J. P.; Marco, M. P.; Lechuga, L. M. Biosens. Bioelectron. 2018, 119, 149–155.
